# Novel Tools for Comprehensive Functional Analysis of LDLR (Low-Density Lipoprotein Receptor) Variants

**DOI:** 10.3390/ijms241411435

**Published:** 2023-07-14

**Authors:** Jacek Jasiecki, Monika Targońska, Anna Janaszak-Jasiecka, Magdalena Chmara, Monika Żuk, Leszek Kalinowski, Krzysztof Waleron, Bartosz Wasąg

**Affiliations:** 1 Department of Pharmaceutical Microbiology, Faculty of Pharmacy, Medical University of Gdańsk, 80-416 Gdańsk, Poland; krzysztof.waleron@gumed.edu.pl; 2 Department of Biology and Medical Genetics, Medical University of Gdańsk, 80-210 Gdańsk, Poland; monika.targonska@gumed.edu.pl (M.T.); monika.zuk@gumed.edu.pl (M.Ż.); 3 Department of Medical Laboratory Diagnostics—Fahrenheit Biobank BBMRI.pl, Medical University of Gdańsk, 80-211 Gdańsk, Poland; anna.janaszak-jasiecka@gumed.edu.pl (A.J.-J.); leszek.kalinowski@gumed.edu.pl (L.K.); 4 Center of Translational Medicine, Medical University of Gdańsk, 80-210 Gdańsk, Poland; magdalena.chmara@gumed.edu.pl; 5 Laboratory of Clinical Genetics, University Clinical Centre, 80-952 Gdańsk, Poland; 6 BioTechMed Centre, Department of Mechanics of Materials and Structures, Gdansk University of Technology, 80-233 Gdańsk, Poland

**Keywords:** LDL receptor, LDLR, low-density lipoprotein, LDL uptake, familial hypercholesterolemia, CRISPR

## Abstract

Familial hypercholesterolemia (FH) is an autosomal-dominant disorder caused mainly by substitutions in the low-density lipoprotein receptor (*LDLR*) gene, leading to an increased risk of premature cardiovascular diseases. Tremendous advances in sequencing techniques have resulted in the discovery of more than 3000 variants of the *LDLR* gene, but not all of them are clinically relevant. Therefore, functional studies of selected variants are needed for their proper classification. Here, a single-cell, kinetic, fluorescent LDL uptake assay was applied for the functional analysis of LDLR variants in a model of an LDLR-deficient human cell line. An LDLR-defective HEK293T cell line was established via a CRISPR/Cas9-mediated luciferase–puromycin knock-in. The expressing vector with the *LDLR* gene under the control of the regulated promoter and with a reporter gene has been designed to overproduce LDLR variants in the host cell. Moreover, an *LDLR* promoter–luciferase knock-in reporter system has been created in the human cell line to study transcriptional regulation of the *LDLR* gene, which can serve as a simple tool for screening and testing new HMG CoA reductase-inhibiting drugs for atherosclerosis therapy. The data presented here demonstrate that the obtained LDLR-deficient human cell line HEK293T-ldlrG1 and the dedicated pTetRedLDLRwt expression vector are valuable tools for studying LDL internalization and functional analysis of LDLR and its genetic variants. Using appropriate equipment, LDL uptake to a single cell can be measured in real time. Moreover, the luciferase gene knock-in downstream of the *LDLR* promotor allows the study of promoter regulation in response to diverse conditions or drugs. An analysis of four known LDLR variants previously classified as pathogenic and benign was performed to validate the LDLR-expressing system described herein with the dedicated LDLR-deficient human cell line, HEK293T-ldlrG1.

## 1. Introduction

Familial hypercholesterolemia (FH; MIM# 143890) is one of the most common monogenic diseases, and is characterized by increased plasma LDL-C (low-density lipoprotein cholesterol) and deposits of cholesterol in peripheral tissues, leading to accelerated atherosclerosis and an increased risk of premature coronary heart disease [1,2]. Substitutions in the *LDLR* gene (low-density lipoprotein receptor; MIM# 606945) are the leading cause of FH. However, in some probands, the FH phenotype is associated with variants of other genes, i.e., *APOB* and *PCSK9* [3,4].

The low-density lipoprotein (LDL) receptor (LDLR) is a membrane glycoprotein with a molecular mass of 160 kDa, that mediates the uptake of lipoprotein particles, mainly LDL, into cells via receptor-mediated endocytosis [5]. The *LDLR* gene, located on chromosome 19p, spans 45 kb and comprises 18 exons, encoding five domains that form the cell-surface LDL receptor. LDLR transcription is regulated in response to cellular demand for cholesterol by the sterol-responsive, element-binding protein (SREBP) that binds to the sterol-regulatory element (SRE) sequence located in the promoter region [6].

To date, more than 3000 different genetic variants on the *LDLR* gene have been described (https://www.ncbi.nlm.nih.gov/clinvar/ (accessed on 5 June 2023)), but not all of them are likely pathogenic or pathogenic [7,8,9,10]. Furthermore, it is accepted that alterations in the different domains of the LDLR have a distinct impact on the receptor structure and function, thus influencing the severity of the clinical phenotype. Based on the impact of genetic alteration on the expression, function, location, and activity of the LDL receptor, *LDLR* variants have been divided into six classes: Class 1—null, no detectable LDLR protein synthesis; Class 2—defective LDLR transport from the endoplasmic reticulum to the Golgi apparatus; Class 3—binding-defective, impaired LDL to LDLR binding; Class 4—internalization defective, no LDLR/LDL internalization because of defective in clathrin-mediated endocytosis (CME); and Class 5—recycling-defective, no LDLR recycling [11,12,13]. Additionally, a sixth class has been proposed, which includes variants incorrectly inserted into the cell membrane [14,15,16].

It has been reported that distinct classes of LDLR variants have been associated with different responses to statins and risks of premature coronary heart disease, i.e., statin treatment of heterozygous patients with Class 2 and Class 5 variants results in a higher percentage decrease in LDL-c than in patients carrying variants of other classes. It has been suggested that more appropriate treatment could be predicted based on the known phenotype of patients carrying LDLR substitutions [17,18,19]. Several methods have been applied and reported to date to study the impact of genetic variants on the LDL level and the expression and activity of LDLR, including measurement of uptake and degradation of 125I or fluorescently labeled LDL, immunoblotting, immunofluorescence microscopy analysis, and flow cytometry [20,21,22]. The most common method to characterize LDLR variants and LDL uptake is an in vitro assay performed in the LDLR-defective Chinese hamster ovary (CHO) cell line (CHO-ldlA7) transfected with vectors encoding LDLR variants [13,23,24,25]. However, there are reports using other cellular and machine learning models to accurately predict the pathogenicity of *LDLR* missense variants [16,26,27,28].

Since CHO cells lack human proteins, studying the human LDLR variants may not reflect an entire milieu of protein–protein interactions that could influence the LDLR variant’s functionality. Here, we have constructed and employed an LDLR-defective HEK 293T human cell line to introduce a vector containing *LDLR* variants, under the control of the inducible promoter. We propose this system as a valuable tool for functional studies of human *LDLR* variants in a human cell model. To test the described system, we performed functional assays with four *LDLR* variants previously described and classified as pathogenic and benign: c.185C>T (p.Thr62Met), c.661G>T (p.Asp221Tyr), c.1216C>T (p.Arg406Trp), c.1322T>C (p.Ile441Thr) [29,30,31,32].

## 2. Results

### 2.1. Construction of Stable Mammalian Cell Line (HEK293T-ldlrG1) for Inducible Expression of LDL Receptor

**Figure 1:**
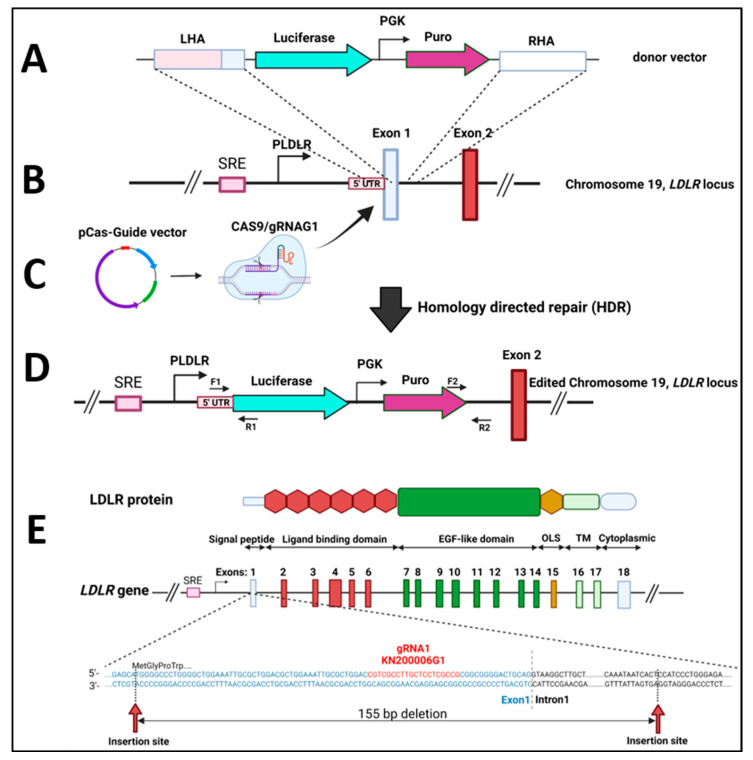
Schematic representation of CRISPR/Cas9 knock-in at human LDLR locus. (**A**) Scheme of the donor vector for gene targeting (# KN200006LP-D). Pink and white rectangles represent the left (LHA) and right (RHA) homologous arms. The turquoise arrow represents the luciferase gene, and the purple arrow represents the coding region of the puromycin gene (Puro). (**B**) Scheme of the human LDLR locus. Blue, green, orange, and red rectangles represent exons, and purple and pink boxes represent the SRE sequence and 5′ UTR, respectively. Black arrows mark promoter regions of both PLDLR and PGK. (**C**) The pCas-Guide vector OriGene (# KN200006G1) and Cas9/gRNA1 complex. (**D**) Targeted allele with homologous recombination. F1 and R2 primers anneal outside of the left and right homologous arms. PCR products were obtained (779 bp and 1453 bp with pairs of primers, F1/R1 and F2/R2, respectively) with templates of genomic DNA from cells with homologous recombination only. (**E**) Schematic representation of LDLR locus and position of the insertion sites of the puromycin functional cassette. The Cas9/gRNA1 DNA cleavage, followed by homologous repair and recombination with the luciferase–puromycin functional cassette, causes 155 bp deletion in exon1 and intron 1. The first amino acids of the LDLR are marked above exon 1. Insertion of the cassette is localized in the first codon of the LDLR. The target sequence for the Cas9/gRNA1 complex is labeled in red. Created with BioRender.com.

For functional studies of the LDLR variants introduced into cells on the vector, switching off endogenous LDLR expression is necessary. The best way to permanently cease LDLR expression is to knock out the gene locus on the chromosome. The gene-editing technology, the CRISPR/Cas9 system, was used to construct the LDLR-deficient HEK293T cell line. The CRISPR/Cas9 knockout/knock-in LDL Receptor (LDLR) Human Gene Knockout Kit from OriGene (# KN200006LP) was used to generate a genomic *LDLR* knockout in HEK293T cells. In brief, the pCas-Guide vector OriGene (# KN200006G1) with the LDLR target sequence (gRNA) near the 5′ end of the *LDLR* gene was introduced together with a donor knock-in vector (# KN200006LP-D) into HEK293T cell line using JetPRIME (Polyplus, Illkirch, France) reagent. The knock-in donor vector contains a luciferase–puromycin functional cassette (3543 bp) between the left and right LDLR homologous arms. After homologous recombination, the luciferase gene is controlled by the native *LDLR* promoter (*PLDLR*), and the puromycin gene is transcribed from the PGK promoter (Figure 1A–D). The transfected cells were passaged several times and then selected with 5 µg/mL puromycin to obtain stable cell clones inserted in the *LDLR* gene luciferase-puromycin cassette, here described as HEK293T-ldlrG1.

### 2.2. Knock-In Screening

**Figure 2:**
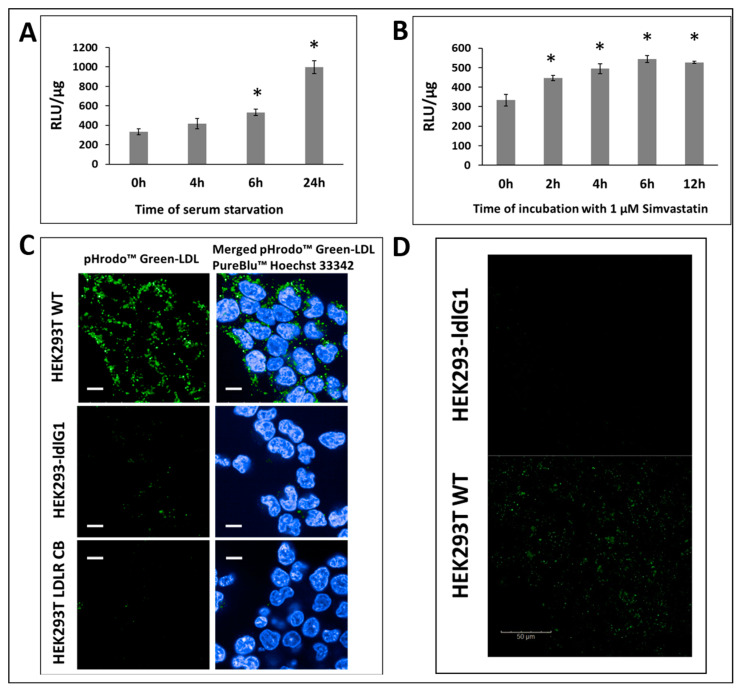
Knock-in screening. (**A**) LDLR promoter–luciferase (PLDLR-Luc) activity assay in HEK293T-ldlrG1 cells. The Luciferase reporter gene was knocked in downstream of the LDLR promoter (PLDLR). The activity of the LDLR promoter was monitored via the luciferase assay in a medium containing 10% FBS (0 h) and after starvation in a serum-free medium for 4, 6, and 24 h. wt HEK293T cells were used as a negative control. (**B**) *LDLR* promoter–luciferase (*PLDLR-Luc*) activity assay in HEK293T-ldlrG1 cells without or after 2, 4, 6, and 12 h of incubation with 1 μM simvastatin. RLU/µg refers to relative luminescence units normalized to µg of total protein in lysates. The asterisks indicate statistically significant results (*p* < 0.05). (**C**) Uptake of pHrodo™ Green-LDL in HEK293 wt, HEK293T-ldlrG1, and *LDLR* knockout HEK293TCB cells from Creative Biogene Inc., Shirley, NY, USA. Scale bar, 10 µm. (**D**) Quantification of LDLR expression via LDL binding assay using fluorescent probe Bodipy FL dye-labeled LDL (green). Scale bar, 50 µm.

The knockout efficiency was evaluated via PCR of the disrupted region containing the LDLR locus on the chromosome. Genomic DNA from puromycin-resistant cells was extracted, and PCR was performed with specific primers annealing to sequences located outside the homologous arms, thus allowing PCR amplification only in the case of the correct homologous recombinant (knock-in). The amplification products (F1/R1 and F2/IR2) were 779 bp and 1453 bp, respectively. DNA products were sequenced to confirm the appropriate knock-in (Figure 1D). As illustrated in Figure 1E, after the specific genome cleavage mediated by CRISPR/Cas9 with gRNA1 and homology-directed repair, the luciferase–puromycin functional cassette is localized in Exon1 of LDLR alleles. The presence of the luciferase-puromycin functional cassette is also manifested by the puromycin resistance of the cells and the luciferase activity. The luciferase activity was monitored in the knock-in and wild-type (WT) cell lysates as described in methods Section 4.3. The presence of the luciferase gene knock-in downstream of the native LDLR promotor (PLDLR) gives promoter–reporter gene fusion that allows the study of the promoter activity regulation under different conditions, i.e., cholesterol starvation or the presence of statins. Moreover, the advantage of such localization of the reporter gene is that the luciferase gene knock-in is in the locus on the chromosome in the natural LDLR gene position, so all potential regulatory elements that are localized; even Mb from the TSS (Transcription Start Site) will be engaged for the such a regulation. We checked the functionality of the *PLDLR*-luciferase promoter fusion by measuring luciferase activity in cells grown in serum-deficient media. The activity of *PLDLR* is upregulated during cholesterol depletion as a function of time (Figure 2A). Moreover, the activity of *PLDLR* is known to be upregulated by statins. Here, we show that the reporter *PLDLR*-luciferase fusion activity is also higher in the presence of Simvastatin in cholesterol-rich media (Figure 2B).

HEK293T cell line has been described in the literature as cells with no or low expression of LDLR [33] and was therefore used in these studies. Although the expression of native LDLR is at a low level in HEK293T, the LDL uptake study in wild-type and LDLR deficient cell lines revealed the presence of a fluorescence-labeled signal of LDL inside of HEK293T and the lack of the signal in HEK293T-ldlrG1 (Figure 2C). This result functionally confirms LDLR deficiency in HEK293T-ldlrG1 cells and the presence of LDLR in wt HEK293T cells. A more sensitive detection method was applied to visualize the presence of the LDLR on the surface of wt HEK293T cells. We used fluorescent-dye-labeled LDL (Bodipy FL-LDL) that binds to LDLR on the cell surface. The fluorescence of cells was monitored for 20 min after the addition of the Bodipy FL-LDL. The signal of the labeled LDL on the cell surface was observed only in HEK293T but not in HEK293T-ldlrG1 cells (Figure 2D).

### 2.3. Construction of Recombinant Expression Vector

**Figure 3:**
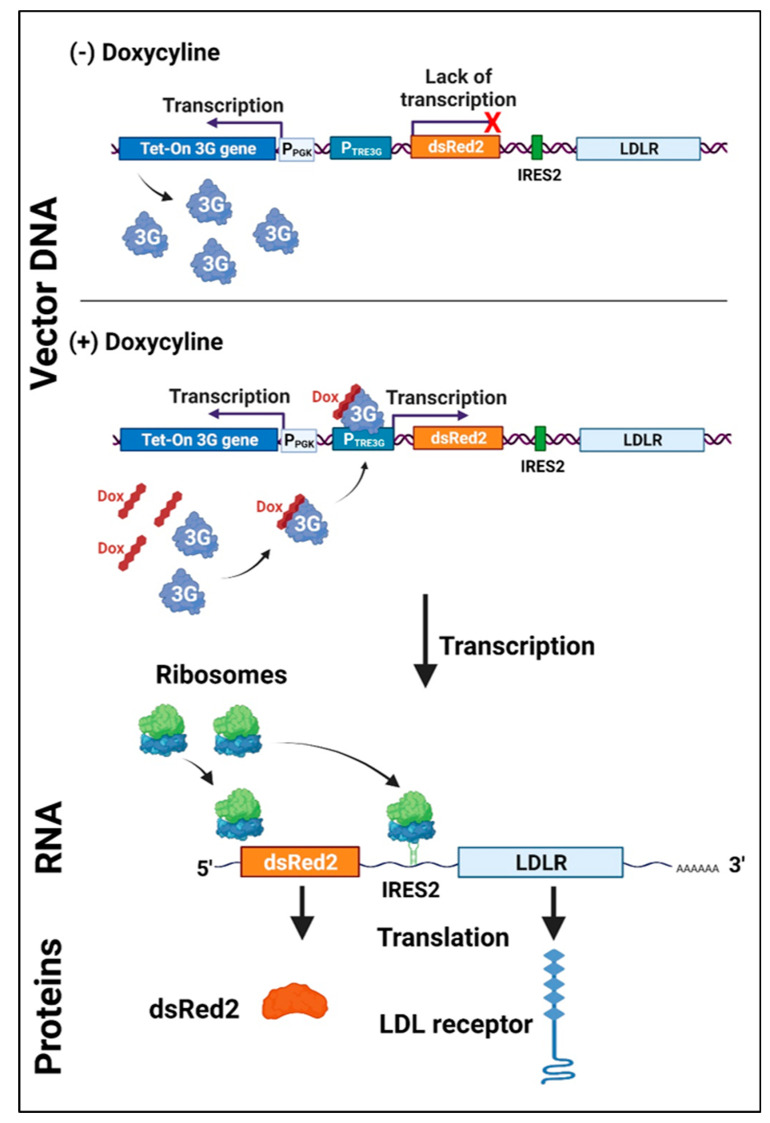
Scheme of the regulation of the LDLR-expressing vector. The Tet-On 3G activator protein is expressed constitutively from the human PGK promoter (PPGK) in the vector but is unable to bind to the TRE3G promoter (PTRE3G) in the absence of doxycycline (Dox). After adding Dox to the culture medium, the activator undergoes a conformational change, binds to PTRE3G, and activates transcription of the bicistronic transcript consisting of the dsRED2 and LDLR cloned downstream. Both proteins, the reporter and the LDLR, are translated from one transcript. Created with BioRender.com.

For the functional study of *LDLR* gene variants, the pTetRedLDLRwt, expressing a full-length LDLR, was constructed. The *LDLR* gene was inserted as a bicistronic construct with a fluorescent reporter protein (DsRed2) under the control of a pTet3G inducible promoter. The inducible promoter was applied to acquire the desired LDLR level on the cell surface. The level of transcription from the promoter and, thus, the level of protein expression was controlled by the concentration of doxycycline. DsRed2 and *LDLR* genes are transcribed in the same single bicistronic transcript, but the downstream LDLR is translated under the control of a viral IRES2 sequence. Such a construct allows monitoring of the LDLR expression level as a function of DsRed2 fluorescence since both proteins are expressed together proportionally (Figure 3). It also enables control of the transfection’s efficiency and selection of the appropriate single cells for functional analysis of LDLR variants.

### 2.4. Expression of LDLR in HEKT-ldlrG1 Cells

**Figure 4:**
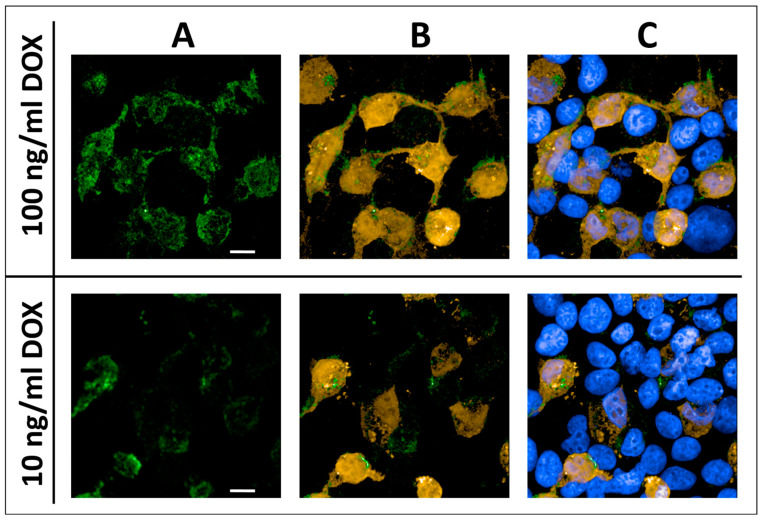
The immunofluorescence analysis of the LDLR and DsRed2 expression from the pTetRedLDLRwt vector in HEK293T-ldlrG1 cells via confocal microscopy. The cells were grown in 100-nM and 10-nM concentrations of the inducer (DOX) for 48 h and then immunostained as described in Materials and Methods Section 4.7. (**A**) Anti-LDLR monoclonal antibody (green). (**B**) Merged signals, anti-LDLR antibody (green) and reporter fluorescent protein DsRed2 (orange). (**C**) Merged signals, anti-LDLR antibody (green), reporter fluorescent protein DsRed2 (orange), and nuclei stained with PureBlu™ Hoechst 33342 (blue). Scale bar, 10 µm.

**Figure 5:**
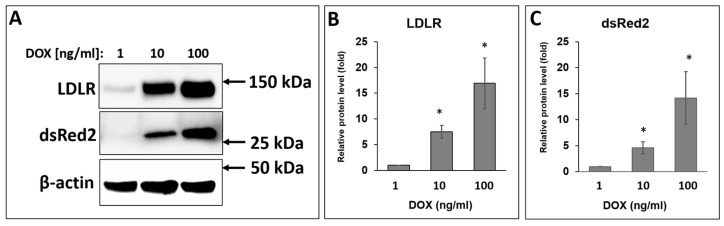
(**A**) Protein overexpression level of LDLR and DsRed2 in HEK293T-ldlrG1 cells transfected with pTetRedLDLRwt vector and analyzed via Western blot. The overexpression was conducted for 72 h in 1-nM, 10-nM, and 100-nM concentrations of doxycycline. Subsequently, cells were lysed, and whole cell extracts (50 μg) were fractioned via SDS-PAGE and processed as described in the Materials and Methods section. The relative protein level was calculated as the LDLR (**B**) or DsRed (**C**) band intensity ratio to that of β-actin (*n* = 3). The asterisks indicate statistically significant results (*p* < 0.05).

The cell line lacking endogenous LDLR, HEK293T-ldlrG1 cells, was transfected with the pTetRedLDLRwt vector carrying the *LDLR* gene. The LDLR expression was assayed via confocal microscopy and Western blotting using a specific anti-LDLR monoclonal mouse antibody. The expression of the LDLR from the TRE3G promoter from the pTetRedLDLRwt in HEKT-ldlrG1 cells was turned on for 48–72 h, using different concentrations of doxycycline (Dox). In fixed cells, the overexpressed LDLR was found at the cell surface (Figure 4A), and the expression level from the vector was monitored via fluorescence of the DsRed2 protein in confocal microscopy (Figure 4B). The presence of the signal from the anti-LDLR antibody was found only in cells expressing the DsRed2 reporter protein. The cell nuclei were stained with Hoechst 33342, visualizing the remaining cells that had not been transfected and lacked the LDLR and DsRed2 signal (Figure 4C). The overexpression level of the LDLR and DsRed2 from the pTetRedLDLRwt vector was also monitored via Western blotting. As shown in Figure 5, the band corresponding to the mature form of LDLR as well as DsRed2 protein are detected in cells transfected with the LDLR-expressing vector. In contrast, no LDLR protein was detected in non-transfected HEK293T-ldlrG1 cells. The intensity of LDLR and DsRed2 signals was proportional to doxycycline concentration (Figure 5A–C).

### 2.5. Functional Analysis of LDLR via Measurement of Labeled LDL Uptake

**Figure 6:**
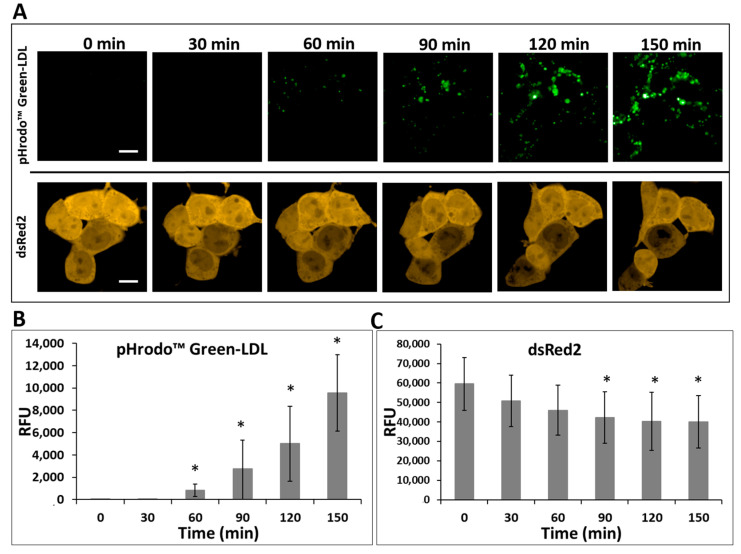
(**A**) Uptake of pHrodo™ Green-LDL in HEK293T-ldlrG1 cells transfected with the pTetRedLDLRwt vector in time intervals, every 30 min. The expression level of the reporter protein DsRed2 was constant during the assay. The labeled LDL (green) was internalized and accumulated by cells in the function of time. Since the pH-sensitive label was used, only the uptaken LDL is visible in the cells. Scale bar: 10 µm. The relative fluorescence level was calculated from three single cells of pHrodo™ Green-LDL (**B**) and dsRed2 (**C**). RFU stands for relative fluorescence unit, (*n* = 6). The asterisks indicate statistically significant results (*p* < 0.05).

An LDL internalization study was conducted to determine the functionality of the overproduced LDLR protein. LDLR-deficient HEK293T-ldlrG1 cells transfected with the pTetRedLDLRwt vector containing recombinant full-length LDLR were assessed for their ability to uptake pHrodo™ Green-LDL. The analysis was performed in real time using confocal microscopy of the single cells. pHrodo Green-LDL is dimly fluorescent at a neutral pH outside cells but fluoresces brightly after endocytosis. This feature allows for a no-wash protocol and enables kinetic measurement of LDL uptake and trafficking in cells. As shown in Figure 6, the labeled LDL, pHrodo™ Green-LDL was cumulated in the cells as a function of time. The more prolonged incubation (extended uptake), the more fluorescent signal of the pHrodo™ Green-LDL was observed in the cells. The expression level of proteins from the bicistronic transcript was monitored via fluorescence of the DsRed2 protein in confocal microscopy.

### 2.6. Proof of Principle Studies. In Vitro Functional Characterization of LDLR Variants

To study the usefulness of the presented tools, we performed functional analysis of four known LDLR variants: c.185C>T (p.Thr62Met); c.661G>T (p.Asp221Tyr); c.1216C>T (p.Arg406Trp); c.1322T>C (p.Ile441Thr) previously described and classified as pathogenic and benign. LDLR deficient HEK293T-ldlrG1 cells were transfected with pTetRedLDLR vectors containing different *LDLR* gene variants, and 48 h after induction with 100 ng/mL of doxycycline (Dox), the expression of LDLR was assayed via immunoblotting with a specific antibody.

**Figure 7:**
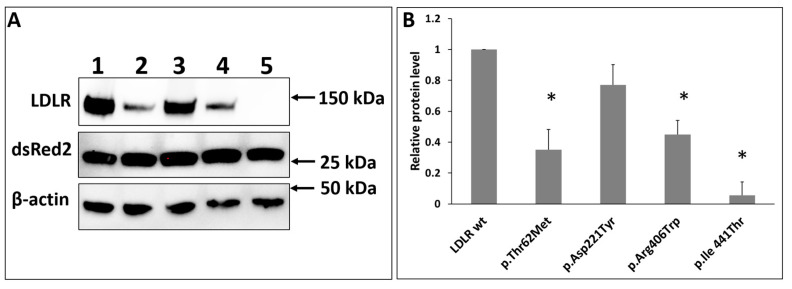
(**A**) Western blot of protein overexpression level of LDLR and dsRed2 in HEK293T-ldlrG1 cells transfected with vectors expressing LDLR wild-type (lane 1), p.Thr62Met (lane 2), p.Asp221Tyr (lane 3), p.Arg406Trp (lane 4), and p.Ile441Thr (lane 5) variants. The overexpression was conducted for 72h, and subsequently, cells were lysed, and whole cell extracts (20 μg) were fractioned via SDS-PAGE, then processed as described in the Materials and Methods section. The relative protein level was calculated as the ratio of LDLR band intensity to that of β-actin (**B**) (*n* = 3). The asterisks indicate statistically significant results (*p* < 0.05).

**Figure 8:**
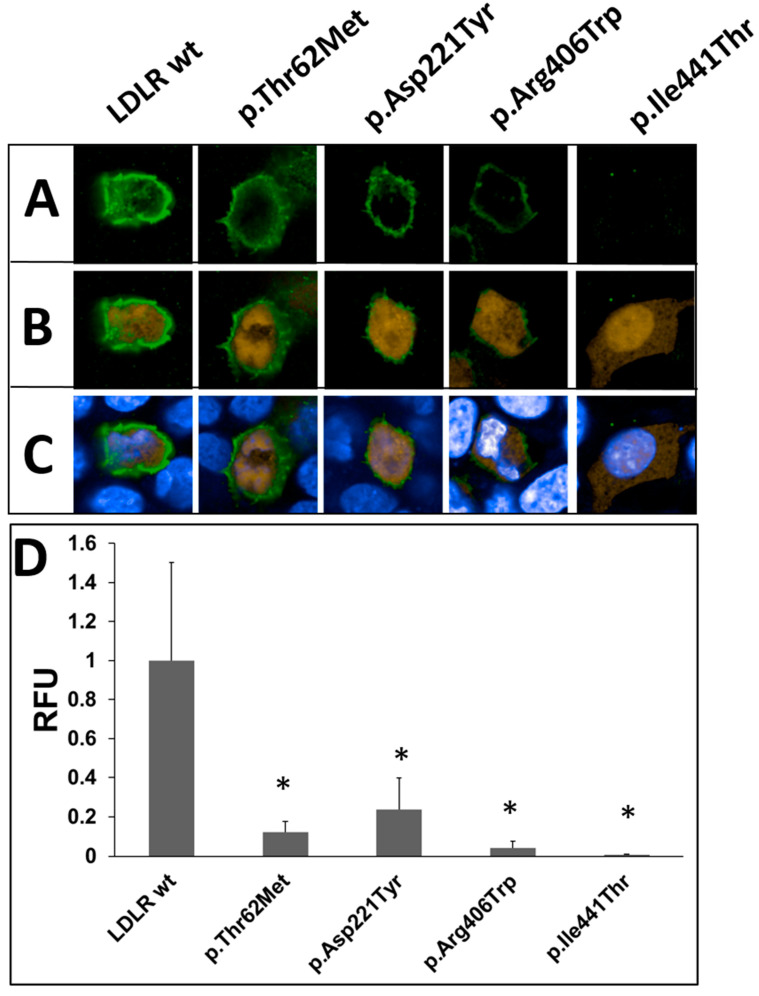
Immunofluorescence analysis of the LDLR and dsRed2 in HEK293T-ldlrG1 cells transfected with vectors expressing wild-type LDLR, p.Thr62Met, p.Asp221Tyr, p.Arg406Trp, and p.Ile 441Thr via confocal microscopy. Cells were grown in 10ng/mL-concentration of the inducer doxycycline (Dox) for 48 h and then immunostained as described in Materials and Methods Section 4.7. (**A**) Anti-LDLR monoclonal antibody (green). (**B**) Merged signals; anti-LDLR antibody (green); and reporter-fluorescent protein, dsRed2 (orange). (**C**) Merged signals; anti-LDLR antibody (green); reporter-fluorescent protein; dsRed2 (orange); and nuclei stained with PureBlu™ Hoechst 33342 (blue). The relative fluorescence (RFU) level was calculated from three single pHrodo™ Green-LDL cells (*n* = 3) (**D**). The asterisks indicate statistically significant results (*p* < 0.05).

**Figure 9:**
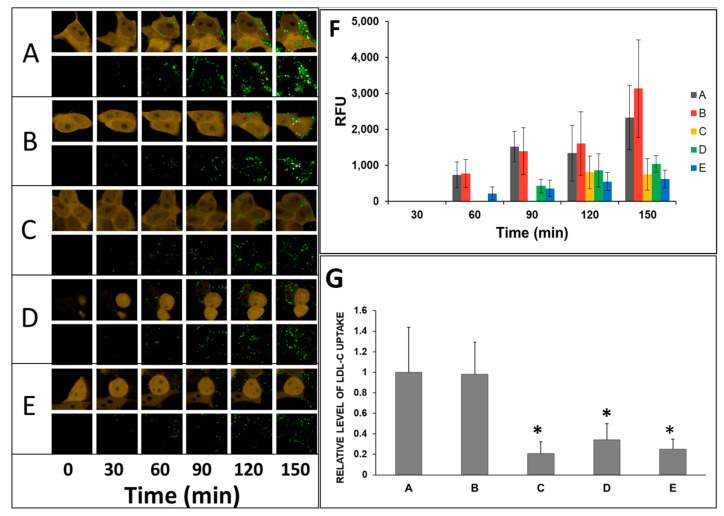
**(A**) Uptake of pHrodo™ Green-LDL in HEK293T-ldlrG1 cells transfected with vectors expressing LDLR wild-type (**A**), p.Thr62Met (**B**), p.Asp221Tyr (**C**), p.Arg406Trp (**D**), and p.Ile441Thr (**E**) in 30-min time intervals. The expression level of the reporter protein, dsRed2, was constant during the assay or slightly dropped due to photobleaching. The labeled LDL (green) was internalized and accumulated by cells as a function of time. Since the pH-sensitive label was used, only the uptaken LDL is visible inside the cells. The relative fluorescence level (RFU) was calculated from three single cells of pHrodo™ Green-LDL (**F**). Characterization of LDLR variants as the function of the uptake of pHrodo™ Green-LDL in time (**G**). Bars represent derivatives of the linear function of the pHrodo™ Green-LDL uptake obtained from values in the graph in Figure 9F. For the LDLR wild-type, the derivative was set as 1 (*n* = 3). The asterisks indicate statistically significant results (*p* < 0.05).

One band corresponding to the mature protein form was detected for wild-type LDLR (Figure 7A, lane 1). As shown in Figure 7A, lane 5, the mature form of the p.Ile441Thr variant was poorly detected via Western blot. The band for the p.Asp221Tyr variant (Figure 7A, lane 3) was comparable in intensity to the wild-type band. For the other two variants, p.Thr62Met and p.Arg406Trp, the signals were three and two times lower, respectively, than those of the wild-type LDLR (Figure 7A, lane 2, 4). Immunofluorescence assays were performed to confirm the presence of the LDLR variants on the cell surface, As shown in Figure 8A, p.Arg406Trp, p.Ile441Thr LDLR variants are poorly expressed on the cell surface; therefore, binding and uptake activities are also affected (Figure 9). The presence of LDLR wild-type and two variants, p.Thr62Met and p.Asp221Tyr, is visible; however, the expression of p.Thr62Met and p.Asp221Tyr is lower than that of wild-type LDLR, similarly to what was observed via Western blotting (Figure 7). The presented results indicate that the four substitutions studied affect protein half-life or have a negative effect on protein translation. An LDL uptake study was performed to investigate the effect of substitutions in the LDLR gene on receptor function. The expression of p.Thr62Met, p.Asp221Tyr, p.Arg406Trp, and p.Ile441Thr was much lower than that of wild-type LDLR (Figure 8B), and LDL uptake activity was significantly reduced, except for in the p.Thr62Met variant. As reported by others, these three variants, p.Asp221Tyr, p.Arg406Trp, and p.Ile441Thr, cause a substantial impairment of LDL binding, inhibiting LDL uptake [31,32]. However, the p.Thr62Met variant, previously described as benign, has LDL uptake activity similar to wild-type LDLR, although cell-surface expression of LDLR surprisingly appears to be 10-fold lower than that of wild-type LDLR.

One possible explanation for this inconsistency is that the specific antibody recognizes this variant with lower affinity because the p.Thr62Met substitution is located in the L1 region, the target epitope for the anti-LDLR antibody used in this study. Other studies also describe the p.Thr62Met variant as non-pathogenic and benign, not affecting LDLR activity [30].

## 3. Discussion

Coronary heart disease (CHD) is one of the most common yearly death causes. FH patients have an increased risk of developing CHD, but this can be prevented by early identification and LDL-lowering therapy with statins. More than 3000 variants of the *LDLR* gene have been described so far, but not all are pathogenic. Interpreting the clinical significance of LDLR variants is a challenge for molecular diagnostics and is essential to providing accurate, personalized treatments. DNA sequencing is preferred for FH diagnosis as it provides unequivocal results. However, it is useful only for identified and described substitutions. Functional studies have only been performed for about 15% of all LDLR variants [8]. For new and undefined LDLR variants, functional analysis is required. Switching from radioisotopic to fluorescence methods of LDL labeling has lowered the cost and provided a safe and accessible tool to characterize LDLR variants.

Here, we have applied a single-cell, kinetic, fluorescent LDL uptake assay to functionally analyze LDLR variants in the LDLR-deficient human cell line model. Since most previously described functional assays are based on an LDLR-deficient Chinese hamster ovary (CHO) cell line (CHO-ldlA7), we decided to adopt a human cell line for this assay. The human cell line is a more accurate model, with the whole proteome and possible protein–protein interactions more similar to the conditions in patients’ cells. The LDLR-defective HEK293T cell line was established via a CRISPR/Cas9-mediated luciferase–puromycin knock-in. HEK293T wt cells express endogenous LDLR at a very low level which is hard to detect via specific antibodies but sufficient to observe LDL uptake. We constructed an LDLR-expressing vector with the gene under the control of the doxycycline-regulated promoter to overproduce LDLR variants in the LDLR-null background. After adding the inducer to the culture medium, the activator undergoes a conformational change, binds to PTRE3G, and activates transcription of the bicistronic transcript consisting of the DsRed2 and LDLR cloned downstream. Both proteins, reporter and LDLR, are translated from one transcript. Therefore, their expression level can be monitored and tuned based on the fluorescence intensity of the reporter protein, DsRed2.

LDL from human plasma complexed with pHrodo Green dye (pHrodo Green-LDL) is a very useful and sensitive tool in studies of LDL uptake through endocytosis and the trafficking of LDL throughout the cell because it is faintly fluorescent at a neutral pH outside of cells, but fluoresces brightly after endocytosis. The Opera Phenix^®^ High-Content Screening System enables the monitoring of the process of LDL uptake via a single cell or group of cells during cell growth every several minutes. This, in turn, allows the determination of kinetic parameters of LDL uptake for individual cells.

SREBP-mediated regulation of LDLR is crucial for the action of statin drugs in lowering plasma LDL-cholesterol levels in individuals at risk for atherosclerosis. Statins inhibit HMG CoA reductase, thus lowering cholesterol production, and this decrease in cholesterol activates SREBPs regulation, thereby increasing the number of molecules of LDLR on cell membranes. SREBPs also increase the amount of HMG CoA reductase, but this does not increase cholesterol synthesis because statins inhibit the enzyme [34]. Statins upregulate the *PLDLR* activity, thus enhancing *LDLR* expression and LDL clearance from the bloodstream. Such responses of *LDLR* promoter to statins can be exploited for pharmacotherapy research and new drug design. *PLDLR*–luciferase chromosomal gene fusion allows the very efficient and precise study of the cell response to new drugs, HMG CoA reductase inhibitors, designed for coronary heart disease.

Our results show that LDLR expression in the host cells can be precisely tuned using a designed pTetRedLDLRwt vector with different inducer concentrations. The designed host cells, the LDLR-defective HEK293T cell line, was established via CRISPR/Cas9-mediated luciferase-puromycin knock-in into the promoter region of the *LDLR* gene that makes the tool to study transcriptional regulation of the *LDLR* gene. It can also serve as a tool for screening and testing new HMG-CoA-reductase-inhibiting drugs for atherosclerosis therapy, as presented in this study, such as simvastatin. The presence of 1 μM of simvastatin in the media increased the activity of the *LDLR* promoter–luciferase fusion. The functional analysis and the level of LDLR expression were measured using immunofluorescence, Western blot, and a single-cell, kinetic, fluorescent LDL uptake assay.

Here, we provide a powerful tool for the functional analysis of LDLR variants and the impact of LDLR mutations on protein activity. The strength of these tools relies on their precision; the expression level of LDLR and the uptake of LDL to a single cell in real time can be measured using appropriate equipment. Moreover, luciferase gene knock-in downstream of the LDLR promotor enables the study of promoter regulation in response to statins and can help in the study of LDLR expression response to new lipid-lowering drugs. To analyze the usefulness of the presented tools in variant classification, proof-of-principle experiments were performed based on functional assays with four variants, previously characterized as LDLR mutations, and classified as pathogenic: p.Asp221Tyr, p.Arg406Trp, p.Ile441Thr, and benign variant, p.Thr62Met. The results indicate that the four substitutions studied affect the protein half-life or negatively affect protein translation. The expression of pThr62Met, p.Asp221Tyr, p.Arg406Trp, and p.Ile441Thr is much lower than that of wild-type LDLR. LDL uptake in all tested variants is significantly reduced, except for the p.Thr62Met variant that has LDL uptake activity similar to wild-type LDLR. On the other hand, as reported by others, three pathogenic variants, p.Asp221Tyr, p.Arg406Trp, and p.Ile441Thr, cause a substantial impairment of LDL binding, inhibiting LDL uptake [31,32].

## 4. Materials and Methods

### 4.1. CRISPR/Cas9 Mediated Knock-In at the Human LDLR Locus

HEK293T cell line was cultured in 40% MEM, 40% F12, 10%William’s E medium supplemented with 10% fetal bovine serum, 2 mmol/L L-glutamine, 100 units/mL penicillin, and 100 μg/mL streptomycin. The CRISPR/Cas9 knockout/knockin LDL Receptor (LDLR) Human Gene Knockout Kit from OriGene (# KN200006LP) was used to generate a genomic LDLR knockout in HEK293T cells. In brief, 1.0 × 106 HEK293T cells were seeded in one well of a six-well plate and co-transfected with 2.5 μg of the pCas-Guide vector OriGene (# KN200006G1) with the LDLR target sequence (gRNA1) near to the 5′ end of the LDLR gene together with 2.5 μg of the knock-in donor vector (# KN200006LP-D) using JetPRIME reagent according to the manufacturer’s instructions. The donor DNA contained left and right homologous arms and luciferase–puromycin functional cassette for homologous vector recombination into the LDLR locus of the HEK293T cell line. After homology recombination, the luciferase and puromycin were controlled by the native *LDLR* promoter, and the puromycin gene was controlled by a *PGK* promoter. The transfected cells were passaged several times and then grown with the selection in 5 µg/mL of the puromycin containing complete media according to the manufacturer’s instructions, to obtain resistant cells containing luciferase–puromycin functional cassette inserted in the LDLR gene. The control human LDLR knockout HEK293T cells were also purchased from Creative Biogene Inc., Shirley, NY, USA.

### 4.2. Knock-In PCR Screening

Genomic DNA from puromycin-resistant cells was extracted using the Tissue & Bacterial DNA Purification Kit (Eurx, Gdańsk, Poland) according to the manufacturer’s protocol. PCR with specific primers (Supplementary Materials) was used to confirm the appropriate genome edition. Using one primer (F1 or R2) in the pairs that anneal to sequences located outside of the homologous arms thus allowed PCR amplification, only in the case of the predicted correct homologous recombinant (knock-in).

### 4.3. Luciferase Activity Assay

For measurement of LDLR promoter activity, HEK293T-ldlrG1 cells were seeded onto a 12-well plate, and after reaching 80% confluence, cells were starved for the indicated time in serum-depleted media supplemented with 0.3% BSA. Luciferase activity was assessed with Luc-Pair™ Duo-Luciferase Assay Kit 2.0 (GeneCopoeia, Rockville, MD, USA), following the manufacturer’s instructions. Briefly, cells were lysed via adding Ly-sis Buffer directly on a 12-well plate (250 µL/well) and incubated for 10 min on a rocking platform. An amount of 20 µL of each cell lysate was transferred in triplicate into the wells of white OptiPlate-96 (Perkin Elmer, Akron, OH, USA), and then 100 µL of FLuc Assay Working Solution was added to each well, mixed and incubated for 3 min. Luminescence was detected via Cytation3 imaging reader (BioTek, Winooski, VT, USA) within 5 min. Luciferase activity was normalized to sample protein concentration and depicted as RLU/µg protein. Results are presented as averages from three independent experiments.

### 4.4. Western Blot Analysis

Cell lysates were prepared via sonication in standard RIPA buffer, and protein concentration was determined via DC protein assay (Bio-Rad, Hercules, CA, USA) according to manufacturer’s instructions. Samples were mixed with 10 µL Laemmli sample buffer containing 5% β-mercaptoethanol (BME), subjected to electrophoresis on Mini-PROTEAN 4–15% precast TGX Stain-Free gels (Bio-Rad, Hercules, CA, USA) and run until the sample front had passed through the gel, approx. 45 min at 200 V. The gel was then stain-free, activated for 45 s, and imaged using the ChemiDoc Touch Imaging system (Bio-Rad, Hercules, CA, USA) and Image Lab software (version 6.0) (Bio-Rad, Hercules, CA, USA). The activated gel was transferred to a PVDF membrane (Bio-Rad, Hercules, CA, USA) using a wet transfer system in transfer buffer (25 mM Tris, 192 mM glycine) for 60 min at 100 V. The PVDF membrane was blocked with gentle agitation in TBST buffer (0.1% Tween 20 and 150 mM NaCl in 10 mM Tris–HCL, pH 7.4) with 3% skim milk. Primary mouse monoclonal antibodies anti-LDLR (1:1000) (Progen Biotechnik GmbH, Heidelberg, Germany), anti-dsRed2 rabbit polyclonal antibody (1:1000) (Takara Bio Inc., Shiga, Japan), and anti-β-actin (1:5000) (ab6276, Abcam, Cambridge, UK) diluted in blocking solution (3% skim milk in TBST) were incubated with the membrane overnight at 4 °C. After washing 3 × 5 min with TBST, the membrane was incubated with anti-mouse IgG horseradish peroxidase conjugate (1:3000, Bio-Rad, Hercules, CA, USA) and washed 3 × 5 min with TBST. The signals were developed using a Clarity Western ECL Substrate chemiluminescence kit (Bio-Rad, Hercules, CA, USA) or SuperSignal West Dura Extended Substrate (Pierce Biotechnology, Rockford, IL, USA) and detected in the ChemiDoc Touch Imaging system and quantified via densitometry with Image Lab 6.0 software (Bio-Rad, Hercules, CA, USA).

### 4.5. Cloning of cDNA of LDLR into Expression Vector

Vector pCMV6-LDLR with a full-length cDNA clone of LDLR was obtained from the Origene (Origene, Rockville, MD, USA). Vectors, pTetOne and pIRES2-DsRed2, were purchased from Takara Bio Inc., Shiga, Japan. To construct the pTetRedLDLRwt, a full-length cDNA of LDLR with two other PCR fragments consisting of reporter gene, *dsRed2*, and Internal Ribosomal Entry Site (IRES2) sequence were inserted between the *Eco*RI and *Pst*I sites of the pTetOne vector (Takara Bio Inc., Shiga, Japan) using the In-Fusion cloning system (Takara Bio Inc., Shiga, Japan) following the method described by Park et al. [35]. In brief, the cDNA of LDLR, the *dsRed2* gene, and IRES2 sequences were amplified using the CloneAmp HiFi PCR Premix (Takara Bio) with specific primers (Supplementary Materials) from pCMV6-LDLR (Origene, Rockville, MD, USA) and pIRES2-DsRed2 (Takara Bio Inc., Shiga, Japan), respectively. The resulting PCR products have short overlapping sequences at both ends, required for an effective In-Fusion cloning method. The In-Fusion^®^ HD cloning system (Takara Bio Inc., Shiga, Japan) was used to ligate three PCR products in the following order: dsRed2, IRES2, and LDLR with linearized pTetOne vector, which was subsequently transformed into *E. coli* Stellar ™ (Takara Bio Inc., Shiga, Japan), as described by the manufacturer. Bacteria were cultured overnight in the presence of ampicillin 50 µg/mL. The resulting vectors were isolated using the NucleoBond^®^ Xtra Midi EF (Macherey-Nagel, Düren, Germany) plasmid DNA isolation kit according to the manufacturer’s instructions. DNA vectors were subsequently sequenced using BigDye™ Terminator v3.1 Cycle Sequencing Kit.

### 4.6. Transfection

Cells were transfected with constructs expressing human LDLR using JetPRIME Transfection Reagent (Polyplus, Illkirch, France) according to the manufacturer’s protocol. DNA and the transfection reagent and buffer were mixed, incubated at room temperature for 10 min, and added to the cells dropwise. The medium was changed 4 h after transfection. The cells were harvested at 72 h post-transfection, and cell lysate was analyzed for protein via immunoblot, immunofluorescence, or LDL uptake assay.

### 4.7. Quantification of LDLR Expression via Immunofluorescence in Cells

Cells were plated in 96-Well Cell CarrierTM-96 ultra plates (PerkinElmer), fixed with 4% paraformaldehyde for 10 min, and washed three times with 1% BSA in PBS. Samples were then blocked with 10% FBS in PBS for 1 h and washed thrice with 1% BSA in PBS. Subsequently, cells were incubated with the anti-LDLR mouse monoclonal antibody (1:100) (Progen Biotechnik GmbH, Heidelberg, Germany) for 16 h at 4 °C, followed by washing and incubation with the appropriate fluorescent Alexa Fluor 488-conjugated secondary antibody (Thermo Fisher Scientific, Waltham, MA, USA). Cells were counter-stained with PureBlu™ Hoechst 33342 Nuclear Staining Dye (Bio-Rad, Hercules, CA, USA). Samples were analyzed via confocal microscopy, as indicated in Section 4.10.

### 4.8. Quantification of LDLR Expression via LDL-Binding Assay

Cells were plated in 96 Well Cell CarrierTM-96 ultra plates (PerkinElmer). After 18 h incubation, the medium was changed and replaced with 50% MEM, 50%William’s E, 0.3% BSA, 100 units/mL penicillin, and 100 μg/mL streptomycin medium deprived of fetal bovine serum (starvation medium), and cells were incubated for 2 h. To determine LDL-LDLR binding, Bodipy FL dye-labeled LDL (Image-iT™ Low Density Lipoprotein Uptake Kit, Bodipy FL, Thermo Fisher Scientific, Waltham, MA, USA) was added (final concentration, 5 µg/mL), and cells were incubated for an additional 20 min at RT. Cells were washed 3× with 1% BSA in PBS and analyzed via confocal microscopy as described in Section 4.10.

### 4.9. LDL Uptake Assay

Cells were plated in 96 Well Cell CarrierTM-96 ultra plates (PerkinElmer). After 18 h of incubation, the medium was changed and replaced by the starvation medium, and cells were incubated for 2 h. To measure the uptake of LDL, cells were incubated with pHrodo™ Green conjugate concentration 5 µg/mL (pHrodo™ Green-LDL) (Thermo Fisher Scientific, Waltham, MA, USA). The cells were immediately transferred to Opera Phenix High Content Screening System with a controlled environment for live cell imaging (37 °C, 5% CO_2_), and images were acquired every 30 min for 2 h, as described in Section 4.10.

### 4.10. Confocal Laser Scanning Microscopy

The images were obtained using Opera Phenix High Content Screening System and Harmony 4.8 software (PerkinElmer, Waltham, MA, USA) with a 63× water immersion objective (NA 1.15). Alexa 488, Bodipy FL dye-labeled LDL, and pHrodo™ Green conjugate signals were visualized with a 488 nm bandpass excitation filter and 500–550 nm bandpass emission filter. DsRed2 signal was visualized with a 561 nm bandpass excitation filter and a 570–630 nm bandpass emission filter. PureBlu™ Hoechst 33342 signal was visualized with a 405 nm bandpass excitation filter and a 435–480 nm bandpass emission filter. At least thirty 16-bit images were acquired for each sample in confocal mode with a resolution of 1080 × 1080 pixels and binning 2. Exposure time and laser power were kept constant (for each type of imaging) across different repetitions of one type of experiment. Images were processed with Harmony 4.8 software (PerkinElmer). Image analysis to quantify the fluorescence intensities was accomplished using the public domain software, ImageJ 1.53q (NIH, Bethesda, MD, USA).

### 4.11. Statistical Analyses

Statistical analyses were performed with Statistica 13.3 software package (TIBCO Software Inc., Tulsa, OK, USA). Results are presented as means ± SD. Group comparisons were tested using Student’s *t*-test. Differences were considered statistically significant and marked with an * when *p* < 0.05.

## Data Availability

Not applicable.

## Supplementary Materials

The following supporting information can be downloaded at: https://www.mdpi.com/article/10.3390/ijms241411435/s1, primer sequences.
