# Supplementary material for: Novel Tools for Comprehensive Functional Analysis of LDLR (Low-Density Lipoprotein Receptor) Variants
Source: Int J Mol Sci. 2023 Jul 14;24(14):11435. doi: 10.3390/ijms241411435 (PMC10379666; doi:10.3390/ijms241411435)
Supplement: Supplementary file 1 [file ijms-24-11435-s001.zip › ijms-2466387-supplementary.pdf]

Supplementary Materials

Primer sequences:

Primers for knock-in screening:
F1- 5'- GATCAGTGTCTATTAGGTGA
R1- 5'- AATGTCCACCTCGATATGTG
F2- 5'- CCATGCATCGATGATATCAG
R2 - 5' - GGACAAGGTGAGGCTCAGAC

Primers used in construction of the pTetRedLDLRwt vector:
DNA template, pIRES2dsRED – PCR fragment 1 - 708 bp with:
DsRed forw: 5' - ACCCTCGTAAAGAATTCACCATGGCCTCCTCCGAGAA
DsRed rev: 5' - GGATCCCGGGCTACAGGAACAGGTGGTGGCG

DNA template, pIRES2dsRED – PCR fragment 2 - 606 bp with:
IRES 2 forw: 5' - TCCTGTAGCCCGGGATCCGCCCCTCTCCCTC
IRES 2 rev: 5' - GGTTGTGGCCATATTATCATCGTG

DNA template, pCMV6-LDLR – PCR fragment 3 -2627 bp with:
IRES LDLR: 5' - GATAATATGGCCACAACCGCGAGCATGGGGCCCTGGGGC
3 LDLR: 5' – CCTCTAGACATATGCTGCAGTCACGCCACGTCATCCTCCAG

Primers used in construction of the LDLR variants expressing vectors

rs376207800 c.185 C>T p.(Thr62Met)
DNA template, pCMV6-LDLR – PCR fragment – 224 bp
IRES LDLR: 5' - GATAATATGGCCACAACCGCGAGCATGGGGCCCTGGGGC
LDLR185R: 5' – GTGACAGACAAGCACATCTCCTGGGACTCA

DNA template, pCMV6-LDLR – PCR fragment – 2433 bp
LDLR185F: 5' – TGAGTCCCAGGAGATGTGCTTGTCTGTCAC
3 LDLR: 5' – CCTCTAGACATATGCTGCAGTCACGCCACGTCATCCTCCAG

rs875989906 c. 661 G>T p.(Asp221Tyr)
DNA template, pCMV6-LDLR – PCR fragment – 703 bp
IRES LDLR: 5' – GATAATATGGCCACAACCGCGAGCATGGGGCCCTGGGGC
LDLR661R: 5' – CAGATTTGTCCTTGCAGTAGGGGCCACCATCA

DNA template, pCMV6-LDLR – PCR fragment – 1956 bp
LDLR661F: 5' – TGATGGTGGCCCCTACTGCAAGGACAAATCTG
3 LDLR: 5' – CCTCTAGACATATGCTGCAGTCACGCCACGTCATCCTCCAG

rs121908043 c.1216 C>T p. (Arg406Trp)

DNA template, pCMV6-LDLR – PCR fragment – 1253 bp
IRES LDLR: 5' – GATAATATGGCCACAACCGCGAGCATGGGGCCCTGGGGC
LDLR1216R: 5' – CTGACCTCGTGCCAGTTGGTGAAGAAGAGG

DNA template, pCMV6-LDLR – PCR fragment – 1404bp
LDLR1216F: 5' – CCTCTTCTTCACCAACTGGCACGAGGTCAG
3 LDLR: 5' – CCTCTAGACATATGCTGCAGTCACGCCACGTCATCCTCCAG

rs879254862 c. 1322 T>C p.(Ile 441Thr)

DNA template, pCMV6-LDLR – PCR fragment – 1362 bp
IRES LDLR: 5' – GATAATATGGCCACAACCGCGAGCATGGGGCCCTGGGGC
LDLR1322R: 5' – CAGGTCAGACCAGTAGGTTCTATTGCTGGCCA

DNA template, pCMV6-LDLR – PCR fragment – 1297 bp
LDLR1322F: 5' – TGGCCAGCAATAGAACCTACTGGTCTGACCTG
3 LDLR: 5' – CCTCTAGACATATGCTGCAGTCACGCCACGTCATCCTCCAG
